# *Periplaneta americana* (Insecta: Blattodea) and organ fibrosis: A mini review

**DOI:** 10.1097/MD.0000000000032039

**Published:** 2022-12-23

**Authors:** Xin Zhou, Meng Yang, Jing Jin, Jie Chen, Zhi Li

**Affiliations:** a Department of Spleen and Stomach Diseases, The Affiliated Traditional Chinese Medicine Hospital of Southwest Medical University, Lu zhou, Sichuan, China; b Department of Pediatrics, The Affiliated Traditional Chinese Medicine Hospital of Southwest Medical University, Lu Zhou, Sichuan, China; c College of Integration of Traditional Chinese and Western Medicine to Southwest Medical University, Lu zhou, Sichuan, China; d Department of Gastroenterology, The First Affiliated Hospital of Naval Military Medical University (Shanghai Changhai Hospital), Shanghai, China.

**Keywords:** biology, fibrosis, mechanisms, *Periplaneta americana*, pharmacology

## Abstract

Fibrosis is the end stage of many chronic inflammatory diseases and eventually leads to organ failure. Periplaneta americana (P. americana) is referred to as “the product of flesh and blood” in traditional Chinese medicine and has a wide range of therapeutic effects. Owing to the growing interest in this insect for its application in the treatment of tissue injury-healing disorders that induce organ fibrosis, it has attracted the interest of researchers. A literature search was performed using core collections of electronic databases, such as PubMed, Web of Science, China National Knowledge Infrastructure, and Wanfang, using the keywords given below and terms such as pharmacological and biochemical details of this insect. P. americana extracts presented a wide range of therapeutic and biological activities, including antifibrotic, antiinflammatory, antioxidative, and tissue repair activities. Emerging evidence suggests that P. americana extracts may improve scarring, pulmonary fibrosis, liver fibrosis, and kidney fibrosis through the regulation of fibroblast activation, cytokine secretion, and deposition of fibrin, indicating the potential role of P. americana as a therapeutic option for organ fibrosis. P. americana is a potential therapeutic agent for treating fibrosis. Further studies are required for a more in-depth characterization of the antifibrogenic mechanism of P. americana prior to its clinical application in the treatment of organ fibrosis. (Fig. 1).

## 1. Introduction

Fibrosis refers to the hardening of organs caused by the excessive deposition of extracellular matrix (ECM) proteins during various chronic inflammatory diseases, resulting in organ failure. The mortality rate from fibrotic diseases has shown an increasing trend over the years, accounting for approximately 45% of the total mortality in developed countries; however, there are no approved antifibrotic therapies.^[1,2]^ Research on developing therapeutic strategies against fibrosis is a key imperative.

*Periplaneta americana (P. americana) (Insecta: Blattodea*) L. is a valuable Chinese medicine material, although it is a notorious pest widely distributed in human habitats.^[3]^ In traditional Chinese medicine (TCM) has been used in the treatment of severe qi- and blood stagnation-induced diseases and various traumas for thousands of years, including tissue sclerosis, abdominal distension, gastric ulcer, malnutrition, and snakebite.^[4]^ Studies have shown that *P. americana* contains a wide range of active ingredients, including amino acids, polysaccharides, nucleosides, and peptides, of which amino acids account for the highest proportion, with a total of 43.17% of free amino acids and 35.37% of essential amino acids after acid hydration.^[5–9]^ Studies have demonstrated a diverse range of therapeutic effects of *P. americana*, such as antifibrotic,^[10,11]^ anti-inflammatory,^[12]^ antioxidation,^[13]^ antitumor,^[5,14,15]^ immunoregulatory effects,^[16]^ and promotion of tissue repair.^[17]^ In recent years, a variety of TCM preparations containing extracts of *P. americana* as the main component have been used in clinical practice, including Kang-fu-xin liquid (KFX), Xin-mai-long injection, Gan-long capsule, and Xiao-zheng-yi-gan tablets (Table 1).

**Table 1:** Overview of clinical application of relevant preparations of *P. americana*.

Name	Character	Efficacy	Attending disease
Kang-fu-xin liquid	Light brown liquid	Improving microvascular perfusion, nourishing yin and generating muscle	Oral: upper gastrointestinal ulcer, adjuvant therapy for pulmonary tuberculosis; external application: burn and scald, snake bite, bedsore, etc
Xin-mai-long injection	Yellow clear liquid	Invigorating qi and promoting blood circulation, promoting yang and diuresis	Adjunctive therapy for chronic congestive heart failure
Gan-long capsule	Yellowish brown to tan powder	Harmonizing the liver and spleen, promoting blood circulation and detoxify	Adjuvant therapy for chronic hepatitis B
Xiao-zheng-yi-gan tablets	Tablet	Alleviating blood stasis, relieving swelling and relieving pain	Adjuvant therapy for primary liver cancer

Recently, the antifibrotic activity of *P. americana* has gradually attracted the attention of researchers and has shown good application prospects in the treatment of organ fibrosis. Continuous exposure to an inflammatory microenvironment contributes to an imbalance in tissue injury healing mechanisms that induce fibrotic products. Numerous studies have demonstrated the antioxidant and anti-inflammatory effects of *P. americana* in the setting of organ inflammation, such as in the skin,^[18]^ lung,^[19]^ liver,^[20]^ and stomach.^[21]^ *P. americana* is referred to as “the product of flesh and blood” (a natural animal drug with nutritional support) in TCM, as it helps in restoring the balance of injury-healing mechanisms that induce fibrotic products, mainly breaking accumulation, promoting blood circulation, nourishing yin, and strengthening muscles by inducing potential therapeutic responses.^[17,22,23]^ These functions are considered as the primitive application of the antifibrosis activity of this insect.^[24,25]^ Emerging evidence suggests that *P. americana* can alleviate organ fibrosis, including scarring, in the lungs, liver, and kidney by regulating fibroblast activation, cytokine secretion and ECM deposition function.^[20,26–28]^ Thus, *P. americana* is a potential candidate for an antifibrotic agent. This review focuses on the antifibrotic effects of *P. americana* and the underlying mechanisms.

## 2. Search strategy

To collect relevant *in vitro* and *ex vivo* experiments, we searched electronic databases including 2 English databases [PubMed, Web of Science (SCI)] and 2 Chinese databases [China National Knowledge Infrastructure and Wanfang]. The search keywords used were “*Periplaneta americana*,” “fibrosis,” “mechanisms,” “biology,” and “pharmacology.” The initial search data were March 25, 2022, and an updated search was conducted on June 22, 2022. A database search was conducted by a single researcher (XZ). All studies published before the date of the search were considered.

### 2.1. Inclusion criteria

Relevant study selection was based on the following inclusion criteria: studies on organ fibrosis; intervention for study: extract or active ingredient of *P. americana*; studies performed *in vitro* or *ex vivo*, including primary cells and/or cell lines; and animal experiments without restrictions to species. Two researchers (XZ and MY) independently assessed the eligibility of the searched articles, and any disagreements were resolved through discussion under the arbitration of a third reviewer (JJ).

### 2.2. Exclusion criteria

Relevant study selection was based on the following exclusion criteria: duplicate studies and overlapping criteria; title and/or abstract of the study failed to meet the inclusion criteria; and editorials, letters, conference abstracts, comments, and opinions.^[29]^

The selected articles were evaluated for information on the dosage form of *P. americana*, route of administration, experimental model, potential mechanism, results, and discussion. After applying inclusion and exclusion criteria, 38 articles were selected (Fig. 2).

**Figure 1.**
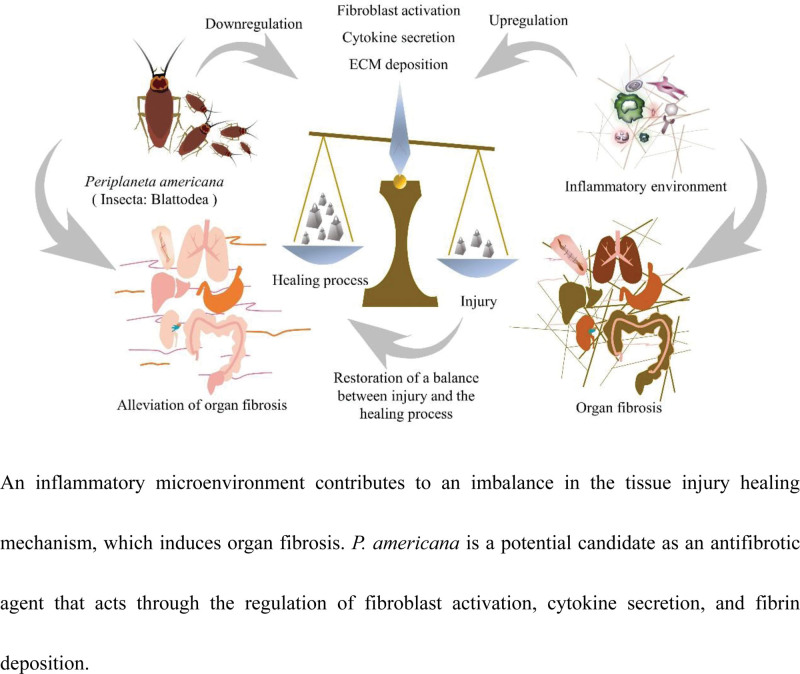
Graphical abstract. An inflammatory microenvironment contributes to an imbalance in the tissue injury healing mechanism, which induces organ fibrosis. *P. americana* is a potential candidate as an antifibrotic agent that acts through the regulation of fibroblast activation, cytokine secretion, and fibrin deposition.

## 3. Pathogenesis of organ fibrosis

The pathophysiology of fibrosis involves the activation of profibrotic fibroblasts, secretion of cytokines, and excessive production and deposition of ECM proteins, such as collagen, elastin, proteoglycan, and glycoprotein.^[30]^ The factors causing fibrosis include wound healing disorders caused by the destruction of the skin ultrastructure (such as cellular mitochondria, endoplasmic reticulum, and desmosomes), oxidative stress response caused by smoke stimulation, persistent infection, repeated exposure to toxins, obesity, genetic factors, and immune diseases. Moreover, the pathological process of fibrosis is complex and involves a cascade of cytokines and transducing signals in response to diverse fibrotic triggers. More specifically, chronic lesions in various organs, such as the skin, lung, liver, and kidney, lead to dyshomeostasis in the injury-healing mechanism that induces fibrotic products. Although there are fibrosis-specific initiators in organs, the molecular mechanisms of fibrosis signaling, such as fibroblast activation, cytokine secretion, integrin receptor activation, upregulation of the transforming growth factor-β (TGF-β) signaling pathway, and ECM deposition, are highly conserved (Fig. 3).

**Figure 2.**
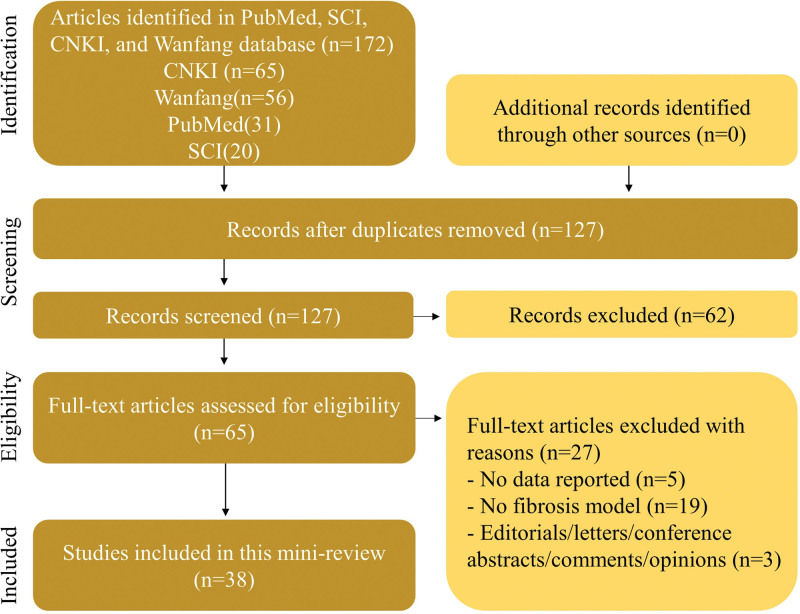
PRISMA flow chart of antifibrotic effect of *P. americana*.

**Figure 3.**
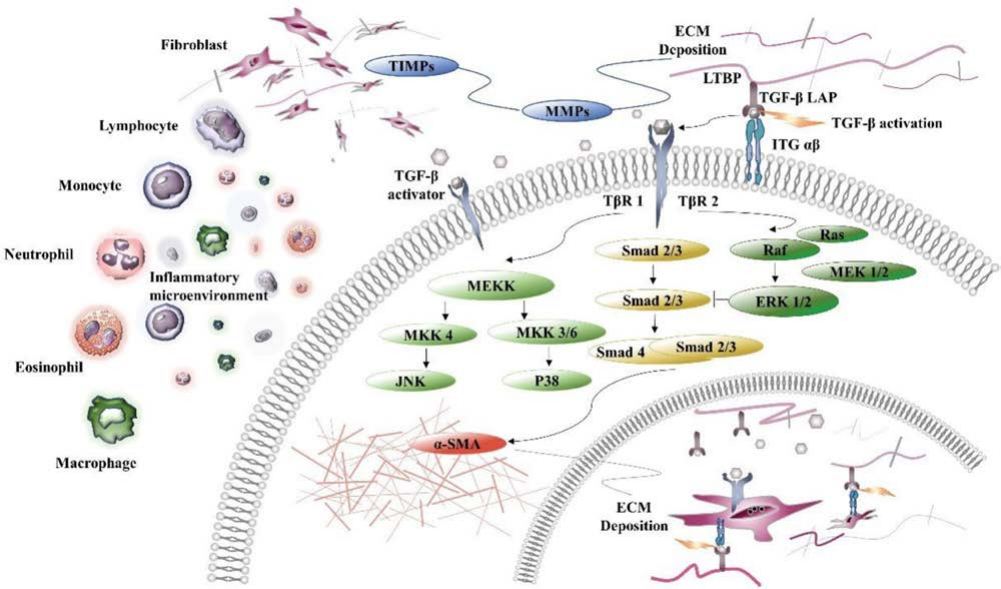
Schematic illustration of the development of fibrosis. A variety of noxious stimuli induce an inflammatory microenvironment that causes the infiltration of macrophages and immune cells. Subsequently, cytokines, especially TGF-β, are released and are highly conserved in the process of organ fibrosis. Activated TGF-β and its ligands bind to integrin receptors and mediate cell–cell and cell–ECM adhesion and signal transmission. Moreover, TGF-β binding to TβR receptors induces nuclear translocation by regulating Smad and MAPK signaling. In this way, fibroblasts are further activated and may begin to release their own autocrine factors and “feed back” some fibrotic signals similar to ECM proteins. TGF-β transforming growth factor β, ECM = extracellular matrix, LAP = latency-associated peptide, LTBP = latent TGF-β binding protein, ITG αβ = integrin αβ, TβR 1/2 = TGF-β receptor 1/2, α-SMA = alpha-smooth muscle actin, TIMPs = tissue inhibitor of matrix metalloproteinases, MMPs = matrix metalloproteinases, MEKK = MAPK kinase, MEK/MKK MAPK kinase, ERK 1/2 = extracellular regulated protein kinase 1/2, JNK = c-Jun N-terminal kinase.

Continuous exposure to various harmful stimuli, including toxins, infectious pathogens, autoimmune reactions, and mechanical stress, induces an inflammatory microenvironment that leads to the infiltration of macrophages and immune cells.^[2,31,32]^ Inflammatory areas have been shown to have prominent infiltrates of lymphocytes, neutrophils, eosinophils, monocytes, and macrophages.^[33,34]^ Lymphocytes, are at the core of the immune response. Stimulation by antigens leads to lymphocyte activation. Neutrophils contain large numbers of lysosomes in the cytoplasm, which activate the phagocytic and digestive functions of cells in the context of tissue damage, acute infection, and malignancy. Eosinophils play an important role in immune response and allergic reactions. These promote inflammatory progression by releasing the granule contents. Macrophages and monocytes are both phagocytes. When monocytes extravasate from blood vessels into connective tissue, they gradually differentiate into macrophages, which are characterized by an increased size, proliferation of endoplasmic reticulum and mitochondria, and increased number of lysosomes with more powerful phagocytic function. The inflammatory microenvironment is an important trigger of regeneration and fibrosis.

The activation of immune cells is presumed to result in the release of early acting cytokines, such as nuclear factor kappa-B (NF-κB), tumor necrosis factor (TNF), and interleukin-6.^[35]^ Subsequent activation and possible phenotypic alteration of structural cells induce the release of other growth factors, including TGF, vascular endothelial growth factor (VEGF), basic fibroblast growth factor, connective tissue growth factor, and platelet-derived growth factor (PDGF).^[36]^ Upon entry into the tissue, these cytokines become further activated and may modulate the differentiation and function of fibroblasts via diverse molecular mechanisms and regulate fibrogenesis. Immune cells are substrates of myeloperoxidase reactions. Peroxidase production is closely related to immune cell infiltration. Reactive oxygen species and proteolytic enzymes not only aggravate the cytotoxic damage response to hypochlorous acid, but also stimulate cytokine production that mediates fibroproliferative responses, including nuclear factor erythroid 2-related factor 2 (Nrf2), NF-κB, TNF-α, and TGF-β.^[37,38]^

TGF-β is a prototypical member of the TGF-β family of growth and differentiation factors. TGF-β is critical for the regulation of the healing process, has an impact on almost every cell type involved, and tips the balance from regulated physiological repair to excessive pathological repair.^[39,40]^ Integrins are transmembrane heterodimeric cell adhesion molecules composed of an α chain and a β chain, in which the α chain is antigen-specific and the β chain is related to signal transduction. Integrins promote cell adhesion and migration but also control the local activation of latent TGF-β contained in the ECM or cell surface reservoirs. Accumulating evidence suggests that the integrin-dependent activation of TGF-β is a key mechanism by which tissue-propagating cells directly circulate and resident immune cells.^[41–43]^ Thus, inflammatory factors, reactive oxygen species, and integrins act as activators of TGF-β receptors. Latency-associated peptide and latent TGF-β-binding protein form large latent complexes. Subsequently, large latent complex cells bind noncovalently to transmembrane protein receptor − integrins on the surface of various cells (including fibroblasts, interstitial cells, and epithelial cells), mediating cell–cell and cell–ECM adhesion and signal transmission. TGF-β ligands bind to TGF-β receptor I (TβR 1) and II (TβR 2) on the cell surface, and activated TβR 1 upregulates Smad2/3 phosphorylation levels and forms a complex with co-mediated Smad4 to induce nuclear translocation via the Smad-dependent classical transduction pathway.^[44,45]^ Alternatively, activated TGF-β can activate the mitogen-activated protein kinase (MAPK) signaling pathways.^[46–49]^ For example, the activator-binding domain of the Ras protein is catalyzed to convert to Ras-GTP, then binds to C-Raf-1 and phosphorylates MAPK kinase (MEK/MKK). This triggers extracellular regulated protein kinase 1/2 (ERK1/2), further upregulating Smad expression. MKK4 and MKK3/6 catalyze the downstream substrates c-Jun N-terminal kinase and p38, respectively, thereby promoting fibroblast activation and collagen deposition.

Moreover, in response to cytokine activation, fibroblast cells undergo further proliferation, and may begin to release their own autocrine factors and “feed back” some fibrotic signals similar to ECM proteins, such as the overexpression of alpha-smooth muscle actin (α-SMA), laminin (LN), fibronectin (FN), and collagen, which triggers the conversion of fibroblasts into a myofibroblast phenotype, leading to further excessive deposition of ECM.^[50–53]^ Matrix metalloproteinases (MMPs) and tissue inhibitors of matrix metalloproteinases (TIMPs) play a pivotal role in both fibrolysis and fibrogenesis.^[54]^ TIMP has been traditionally thought to control ECM proteolysis through direct inhibition of MMP-dependent ECM proteolysis, which suggests that increased TIMP levels result in ECM accumulation, whereas loss of TIMP leads to enhanced matrix proteolysis.^[55,56]^

Therefore, targeting myofibroblast proliferation, regulating cytokine secretion, and reducing ECM deposition are potential strategies to alleviate fibrosis.

## 4. Characteristics of *P. americana*

Traditional Chinese medicine “cockroach” was first published in Shennong’s Herbal Classic and has a long history of use. According to TCM, cockroaches are salty in taste, cold in nature, and act on the meridians of the liver, spleen, and kidney. The book also mentioned a wide range of therapeutic effects of cockroaches, such as breaking accumulation, promoting blood circulation, nourishing yin and strengthening muscles, and relieving swelling and detoxification.^[4]^ The administration of cockroaches involves the consumption of a soup that has been boiled with the dry powder of the whole insect or directly as a poultice applied to the surface of a patient’s wound,^[23]^ and has been used to alleviate severe qi- and blood stagnation-induced diseases and various traumas, including tissue sclerosis, abdominal distension, gastric ulcer, malnutrition, and snakebite. Even today, people in Southwest China still treat wounds with mashed cockroaches to promote wound healing and inhibit scarring.^[57]^ Currently, nutritional interference is considered an option for improving fibrosis,^[30]^ which somewhat coincides with the viewpoint of TCM, because cockroaches, also known as “the product of flesh and blood” (a natural animal drug with nutritional support), can break the accumulation and generate muscle, thereby promoting the quality of tissue repair.^[23]^ *P. americana* belongs to the phylum Arthropoda, class Insecta, order Cockrobiales, and family Cockrobiaceae. *P. americana* may act on fibroblast activation, cytokine signaling, and fibrin component deposition. Evidence has shown that *P. americana* may accelerate acute wounds by increasing the number of blood vessels and regulating the levels of angiogenesis-related cytokines (α-SMA, VEGF, and CD31).^[58]^ Jie and Li et al demonstrated that *P. americana* extracts regulate fibroblast migration and proliferation.^[11,59]^ Moreover, *P. americana* alleviates the deposition of ECM in the late stage of tissue healing in a way that promotes tissue repair and avoids fibrosis formation.^[20,60]^ *P. americana* has great potential for application in full-thickness wound healing. KFX promotes the quality of wound healing by promoting the ultrastructural repair of dermal cells, such as the mitochondria, endoplasmic reticulum, and desmosomes.^[61]^ The colonic mucosa epithelium layer, crypt, muscle layer mucosa, and submucosa were also well repaired after KFX treatment in ulcerative colitis mice.^[62]^ Collectively, these lines of evidence indicate the potential role of *P. americana* as a therapeutic agent for organ fibrosis.

The medicinal value of *P. americana* has also been recognized. Several pharmacological studies have reported the active ingredients in *P. americana*, such as amino acids such as leucine, alanine, proline, and tyrosine,^[63–66]^ nucleoside components such as uracil, inosine, and hypoxanthine,^[67–69]^ and polyol components such as polyol, glycerol, and peptide polyols such as cyclo- (Tyr-Gly).^[70]^ Moreover, 17 compounds have been identified in *P. americana.*^[71]^ Recently, Zhu^[72]^ and Yan^[71]^ reported 8 novel compounds isolated from *P. americana*, including 2-(4′-methyl-3′-pentyloene)-6-hydroxymethyl-10-methyl-12-hydroxyl-(2,6,10)-triendodecanic acid (1), arbutin (2), 4-benzoxy-3-methopyranoside acid (3), (E)-3-hexenyl-β-D-glucopyranoside (4), periplanamides A (5), periplanamides B (6), periplanpyrazine A (7), and salicylic acid methyl ester (8). (Table 2) The studies by Zhu et al note that compound (1) belongs to diterpenoids, whose anti-inflammatory,^[73]^ antioxidant,^[74]^ and antitumor^[75]^ properties have been demonstrated. Compounds (2) and (4) are aminoglycosides, of which (2) responds to inflammation and ulcer lesions,^[76]^ whereas (4) exhibits mild cytotoxicity against tumor cell lines.^[77]^ Compound (3) is a phenolic acid with strong antioxidant capacity.^[78]^ According to Yan’s research, compounds (5) and (7) have similar molecular structures. Compound (7) can upregulate the proliferative activity of human dermal fibroblasts; therefore, it is advantageous for promoting wound healing. Compound (6) promotes angiogenesis in human umbilical vein endothelial cells. Finally, the newly discovered compound (8) was very similar to salicylic acid in structure, except for the additional methoxy groups,^[71]^ and had significant analgesic^[79]^ and anti-inflammatory^[80]^ effects. With the increasing identification of active compounds, the pharmacological effects of *P. americana* will be explored further.

**Table 2:** Compounds newly identified from *P. americana.*

NO.	Compound	Ion Peak	*m/z*	M.F.	Reference
(1)	2-(4′-methyl-3′-pentene)-6-hydroxymethyl-10-methyl-12 -hydroxyl-(2,6,10)-triendodecanic acid	[M + H] +	337.0458	C_20_H_32_O_4_	^[72]^
(2)	Arbutin	[M + H] +	273.0745	C_12_H_16_O_7_	
(3)	4-benzyloxy-3-methoxybenzoic acid	[M + H] +	259.0785	C_15_H_14_O_4_	
(4)	(E)-3-hexenyl-β-D-glucopyranoside	[M + H] +	263.1840	C_12_H_22_O_6_	
(5)	Periplanamides A	[M − H] −	374.1248	C_19_H_21_NO_7_	^[71]^
(6)	Periplanamides B	[M + Na] +	264.0845	C_11_H_15_NO_5_	
(7)	Periplanpyrazine A	[M − H] −	187.0511	C_11_H_10_N_2_O_2_	
(8)	Salicyluric acid methyl ester	-	-	-	

M.F. = molecular form.

## 5. Antifibrogenesis properties of *P. americana*

Owing to the strong anti-inflammatory and antioxidative effects of *P. americana*, some studies have investigated its antifibrogenic effects. *P. americana* has been shown to exert antifibrotic effects through TGF-β/Smad signaling, Shh (Sonic Hedgehog) signaling, Wnt/β-catenin signaling, NF-κB signaling, and the Nrf2/HO-1 (heme oxygenase-1) cascade, which are related to its anti-inflammatory, antioxidant, apoptosis-inductive,^[81,82]^ and gene regulatory effects.^[83]^ They have been shown to inhibit fibroblast activation, cytokine secretion, and deposition of fibrin components. The effects and major mechanisms of *P. americana* extract on organ fibrosis are summarized in Table 3.

**Table 3:** Effects of *P. americana* extracts on organ fibrosis.

Organ	Animal model	Agent	Dose/duration	Activity	Reference
Skin	Mechanical injury scar-induced mice	Ethanol extract: PAE	62.5, 125, 250 g/kg; ext.; 3, 7, 14 d	Facilitating wound repair; reducing collagen content; downregulating CD 68, VEGF, and bFGF	^[86]^
	Scald scar- induced mice	Aqueous extract	0.327, 0.490, 0.653 g/kg; ext.; 5, 10 d	Promoting repair and avoiding scar; regulating TGF-β1 release; inhibiting inflammatory factors release	^[88]^
	Chronic wound-induced rats	Ethanol extract: KFX	0.2 mL/cm^2^; ext.; 5, 7, 11 d	Promoting repair and avoiding scar; inhibiting the deposition of ECM; regulating the expression of Col I/III	^[60]^
	Hypertrophic Scar-induced rabbits	Ethanol extract: KFX	0.5, 2.5, 5 mg/kg; ext.; 28 d	Improving hypertrophic scars by inhibiting hyperproliferation of fibroblasts; downregulating Shh signaling factors (including Shh, Patch, Smo and Gli-1)	^[91]^
Pulmonary	Bleomycin-induced mice	Ethanol extract: KFX	2.5, 5, 10 mL/kg; i.g.; 21 d	Improving pulmonary fibrosis; regulating the dynamic balance between MMPs and TIMPs (including MMP1, MMP3, MMP9 and TIMP-1); downregulating α-SMA, Col I/III; affecting the cycle of fibroblasts by inhibiting Cyclin D1 and promoting CKIs	^[97]^
	Bleomycin-induced rats	Extract: ML-HB	30, 60, 120 mg/kg; i.g.; 30, 45, 60, 75 d	Ameliorating pulmonary fibrosis; reducing TNF-α; inhibiting TGF-β1, α-SMA, Hyp, and col I	^[98,99]^
	Lung’s Oxidative stress-induced mice	Ethanol extract	0.9, 2.7 g/kg; i.g.; 16 d	Improving oxidative stress; reducing MDA and NO; upregulating GSH-Px	^[102]^
Liver	Immune liver fibrosis-induced rats	Extract: thesticky sugar glycine	0.5, 1.25, 2.5 %; i.g.; 28 d	Alleviating liver pathological damage; inhibiting inflammatory factors release (NF-κB, IL-6, ICAM-1); downregulating TGF-β1/TIMP signaling	^[104]^
	Carbon tetrachloride-induced rats	Ethanol extract: KFX	3.1, 6.2, 12.5 mL/kg, i.g.; 28 d	Improving experimental hepatic fibrosis; reducing liver fibrosis markers (HA, LN, PIIINP, Hyp, col III/IV, α-SMA); regulating TGF-β1/Smads and Wnt/ β-catenin signaling pathways	^[106]^
	Carbon tetrachloride-induced rats	Extract: PA-B	30, 60, 120 mg/kg; i.g.; 28 d	Alleviating hepatic oxidative stress; downregulating Nrf2/HO-1 signaling pathway; inhibiting HA, LN, PC-III, Col-IV and MDA; upregulating GSH-Px, CAT, and T-SOD	^[108]^
	Carbon tetrachloride-induced rats	Extract: PA-B	30, 60, 120 mg/kg; i.g.; 28 d	Reducing liver tissue lesions in experimental hepatic fibrosis; inhibiting collagen deposition; regulating TGF-β1/Smad and Bel-2/Bax signaling pathways	^[110]^
Kidney	Unilateral ureteral ligation-induced rats	Ethanol extract: KFX	0.5, 1, 2 mL/kg; i.g.; 7, 14, 21 d	Preventing renal tubulointerstitial fibrosis; affecting TGF-β1/Smad signaling pathway by inhibiting TGF-β1 and Smad3 while promoting Smad7	^[112]^
	Unilateral ureteral ligation-induced rats	Ethanol extract: KFX	5, 10, 20 mL/kg; i.g.; 7, 14, 21 d	Improving renal function; upregulating HGF, MMP-9, and downregulating TIMP-1	^[113]^
Intestinal	Inflammatory bowel fibrosis-induced mice	Ethanol extract	5, 10, 20 mL/kg; i.g.; 40 d	Inhibiting the formation of intestinal fibrosis by regulating TGF-β1/Smad signaling; reducing TGF-β1, Smad2/3 and phospho-Smad2/3 while promoting Smad7; inhibiting α-SMA and Col I	^[114]^
Stomach	Gastric ulcer scar-induced mice	Ethanol extract	10 mL/kg; i.g.; 14 d	Preventing the formation of pathological scar in chronic gastric ulcer; optimizing fibroblast function by inhibiting GH secretion and Hyp synthesis	^[115]^

Ext. = external use, VEGF = vascular endothelial growth factor, bFGF = basic fibroblast growth factor, TGF-β1 = transforming growth factor β1, KFX = kang-fu-xin liquid, Col I/III/IV = collagen type I/III /IV, ig = intragastric administration, MMP1/3/9 = matrix metalloproteinase 1/3/9, TIMP-1 = tissue inhibitor of matrix metalloproteinase-1, α-SMA = alpha-smooth muscle actin, CKI = cyclin-dependent kinase inhibitor, TNF-α = tumor necrosis factor-α, Hyp = hydroxyproline, MDA = malondialdehyde, NO = nitric oxide, GSH-Px = glutathione peroxidase, NF-κB = nuclear factor kappa-B, IL-6 = interleukin- 6, ICAM-1 = intercellular cell adhesion molecule-1, HA = hyaluronic acid, LN = laminin, PC III = procollagen III, PIIINP = type III procollagen amino peptide, CAT = catalase, T-SOD = total superoxide dismutase, Nrf-2 = nuclear factor erythroid 2-related factor 2, HO-1 = heme oxygenase-1, Bcl-2 = B-cell lymphoma-2, Bax Bcl-2 = associated X protein, HGF = hepatocyte growth factor, GH = growth hormone.

### 5.1. *P. americana* and hypertrophic scars

Severe trauma, endocrine disorders, and genetic factors can impair skin repair, leading to hypertrophy. Scar tissue is not a perfect replacement for preinjury tissue because scar tissue exhibits reduced tension and impaired nutrient metabolism.^[84]^ Many studies have confirmed that excessive proliferation of myofibroblasts, disorganization of collagen, and destruction of the ultrastructure of skin cells (such as the mitochondria, endoplasmic reticulum, and desmosomes) are the main pathological features of scars.^[61,85,86]^ These are all associated with the abnormal function of fibroblasts. Thus, improving fibroblast function is a potential mechanism for inhibiting scar formation.

P. americana extracts Ento-A, Ento-B, Ento-C, Ento-D, and Ento-E inhibited the proliferation of CCC-ESF-1 (human embryonic skin fibroblasts) in a time-dependent manner.^[85]^ *P. americana* extract affects wound repair via a variety of mechanisms, including TNF, NF-κB signaling, MAPK, and AMPK signaling.^[87]^ TGF-β1 plays a key role in fibroblast proliferation, differentiation, and migration. Yang et al found that the aqueous extract of *P. americana* promotes the repair of burn wounds while inhibiting scarring owing to its interaction with TGF-β1 release at various stages of the repair process.^[88]^ Another ethanol extract, PAE, reduced collagen fiber content by downregulating CD 68, VEGF, and basic fibroblast growth factor.^[86]^ Moreover, lncRNAs play an important biological role in wound repair. It may regulate the expression of inflammatory factors, such as TNF-α and IL-6, through lncRNAH19 to promote tissue repair and minimize scar formation.^[89]^

KFX, a single preparation of an ethanol extract of *P. americana*, has been found to improve the quality of wound healing. In refractory healing wounds, KFX improves the quality of wound healing by promoting the ultrastructure of dermal cells, such as mitochondria, endoplasmic reticulum, and desmosomes.^[61]^ Moreover, Smads are classical downstream transduction molecules of TGF-β1 signaling that regulate inflammatory signaling in dermal cells,^[90]^ and KFX accelerates the wound inflammatory response by downregulating Smad6 and upregulating Smad9 expression.^[61]^ KFX can inhibit ECM deposition in the late stage of wound healing by regulating the expression of collagen production to promote repair and avoid scar formation.^[60]^ Moreover, KFX inhibits the proliferation of fibroblasts in hypertrophic scars by inhibiting Shh signaling, which is an essential signal transduction pathway in long-term Wnt-activated cells.^[91,92]^ In further studies, Chen et al found that the KFX-treated group had high expression levels of *Ereg*, *Gli2*, and *Tgm3*, as assessed by RNA sequencing.^[93]^ Collectively, these lines of evidence suggest that *P. americana* has the potential to ameliorate scarring.

### 5.2. *P. americana* and pulmonary fibrosis

Pulmonary fibrosis is the end-stage of most chronic lung diseases and is characterized by the initiation of fibroblast recruitment, resulting in excessive ECM deposition.^[94]^ Notably, inflammatory mediators are catalysts that continuously advance the process of pulmonary fibrosis.^[95]^ Therefore, the inhibition of inflammatory mediators and downregulation of specific fibrotic factors may be an effective therapeutic strategy.

In an *in vitro* experiment, *P. americana* extract effectively inhibited the activation of MRC-5 while reducing specific fibrosis factors, including α-SMA, LN, FN, connective tissue growth factor, and Col I/III.^[96]^ The researchers then used primary lung fibroblasts and pointed out that *P. americana* extract may regulate the dynamic balance between MMPs and TIMPs, such as MMP1, MMP3, MMP9, and TIMP-1, which results in ECM degradation and deposition. In addition, it can affect the cycle of primary lung fibroblasts by inhibiting cyclin D1 and promoting cyclin-dependent kinase inhibitors.^[97]^ In in vivo experiments, an extract of *P. americana* (ML-HB) ameliorated bleomycin-induced pulmonary fibrosis by inhibiting the expression of TNF-α, TGF-β1, and hydroxyproline.^[98,99]^ TNF-α is a proinflammatory cytokine that promotes collagen production in addition to stimulating neutrophil and eosinophil infiltration to release lysosomal enzymes.^[100]^ Hyp is a unique amino acid in collagen, accounting for approximately 13% of the total collagen amino acids, and can be used as an indicator of connective tissue degradation.^[101]^ In addition, some extracts exhibited the ability ameliorated the oxidative stress response in the lungs by regulating oxidative stress markers, for example, by downregulating malondialdehyde and nitric oxide and upregulating glutathione peroxidase.^[102]^ These data illustrate multiple mechanisms by which *P. americana* may improve pulmonary fibrosis.

### 5.3. *P. americana* and hepatic fibrosis

Liver cirrhosis often develops in association with hepatic fibrosis because of chronic liver disease. Therefore, exploring potential treatments for hepatic fibrosis is a key research imperative. ML-HB showed a good inhibitory effect on the proliferation of rat hepatic stellate cells (HSC-T6).^[103]^ The sticky sugar glycine, an extract of *P. americana*, protects against liver function damage in immune liver fibrosis and inhibits fibrosis.^[104]^ In addition, there may be a link between its mechanism and downregulation of TGF-β1/TIMP-1 signaling, as well as inhibition of the release of inflammatory factors, including NF-κB, IL-6, and intercellular cell adhesion molecule-1. Intercellular cell adhesion molecule-1 is a transmembrane glycoprotein that plays key roles in leukocyte migration and activation.^[105]^ In a study by Gao et al, KFX was found to inhibit the expression of liver fibrosis markers, including hyaluronic acid, type III procollagen amino peptide, FN, α-SMA, and Col III/IV, by regulating TGF-β1/Smad and Wnt/β-catenin signaling and, more specifically, by downregulating the expression of β-catenin and upregulating the expression of Axin and glycogen synthase kinase 3β.^[106]^ These results suggest that KFX may regulate the “crosstalk” between multiple signaling pathways during the development of liver fibrosis.

Professor Xiao’s research team conducted a series of studies on *P. americana* extract PA-B, which is thought to specifically resist liver fibrosis. PA-B significantly inhibited the proliferation and migration of HSC-T6 cells in vitro by downregulating TGF-β1/Smad signaling. PA-B reduced collagen deposition in the liver interstitium by inhibiting the expression of Col I, Col III, and α-SMA. In addition, PA-B may induce apoptosis in HSC-T6 cells by lowering mitochondrial membrane potential. Alternatively, decreased B-cell lymphoma-2 (Bcl-2) expression, and conversely, increased Bcl-2 associated X protein and caspase-9 expression, also indicate that PA-B activates the apoptosis of HSC-T6 cells.^[107]^ Second, PA-B was found to ameliorate the oxidative stress response in the liver. Nrf-2/HO-1 cascade signaling-related factors are potential targets of PA-B, including Nrf2, NADH quinone oxidoreductase 1, HO-1, and Kelch-like ECH-associated protein 1,^[108]^ which protect against hepatic injury via anti-inflammatory, antioxidant, antiendoplasmic reticulum stress, and antiapoptotic properties.^[109]^ The study also found that PA-B modulated the expression of hyaluronic acid, LN, procollagen III (PC-III), Col-IV, and malondialdehyde while increasing glutathione peroxidase, catalase, and total superoxide dismutase.^[108]^ Finally, in the process of experimental liver fibrosis, PA-B was found to inhibit collagen synthesis and secretion by regulating TGF-β1/Smad and Bel-2/Bcl-2-associated X protein signaling.^[110]^In addition, they conducted an *in vivo* experiment to compare the efficacy of PA-B after gastric and intestinal administrations. The results suggest that both administration methods significantly improved experimental liver fibrosis, but there was no significant difference in efficacy.^[111]^ This is the first study to explore the efficacy of different formulations of PA-B. These findings suggested that *P. americana* alleviates hepatic fibrosis.

### 5.4. *P. americana* and renal fibrosis

Renal fibrosis is an irreversible condition that results from all chronic kidney diseases. Studies investigating the effect of *P. americana* on renal fibrosis are in their preliminary stages. However, KFX exhibited a renoprotective effect by alleviating renal fibrosis induced by unilateral ureteral ligation in a dose-dependent manner, and the mechanism was related to the regulation of the TGF-β1/Smad signaling pathway transducers, including TGF-β1, Smad3, and Smad7.^[112]^ Subsequently, Huang et al conducted a preliminary exploration that suggested that upregulation of hepatocyte growth factor and MMP9 and downregulation of TIMP-1, which reduces nephritic cell infiltration and fibrous tissue deposition, may be potential targets of KFX in renal fibrosis.^[113]^

### 5.5. *P. americana* and fibrosis in other organs

KFX was found to ameliorate chronic inflammation-induced intestinal fibrosis by regulating TGF-β1/Smad signaling and, more specifically, by reducing TGF-β1, Smad2/3, and phospho-Smad2/3 while promoting Smad7.^[114]^ Abnormal proliferation of fibroblasts is a feature of the pathological scars in gastric ulcers. In a previous study, KFX was shown to optimize fibroblast function by inhibiting growth hormone secretion and Hyp synthesis, thereby improving the pathological scar of gastric ulcers.^[115]^

## 6. Conclusions

*P. americana* has a wide range of therapeutic effects in TCM. Advances in modern pharmacological research will help to unravel the potential targets of *P. americana*. Available evidence suggests that *P. americana* extracts can alleviate fibrosis in multiple organs by regulating fibroblast activation, cytokine signaling, and fibrin component deposition. Thus, *P. americana* is a potential therapeutic agent for fibrosis. However, current research on the effect of *P. americana* in improving organ fibrosis is at an experimental stage. More importantly, there is a paucity of studies on its kinetics, metabolism, and toxicity. Therefore, further studies are required for a more in-depth characterization of the antifibrogenic mechanism of *P. americana* prior to its clinical application in the treatment of organ fibrosis.

## Author contributions

XZ (xinzreserch@126.com) and MY (ym1993928@126.com) wrote the main manuscript and prepared the figures and tables; JJ (jinjings1981@126.com) offered professional advices for the article; ZL (lizhi-swmu@126.com) and JC (jiechen0115@sohu.com) coordinated the writing and made the overall revisions to the article. All authors have read and approved the final manuscript.

**Conceptualization:** Xin Zhou.

**Data curation:** Xin Zhou, Meng Yang, Jing Jin.

**Formal analysis:** Meng Yang.

**Funding acquisition:** Jie Chen.

**Methodology:** Xin Zhou, Meng Yang, Jing Jin.

**Project administration:** Jie Chen.

**Software:** Xin Zhou, Meng Yang, Jing Jin.

**Supervision:** Jie Chen.

**Visualization:** Xin Zhou, Meng Yang.

**Writing—original draft:** Xin Zhou, Meng Yang.

**Writing—review and editing:** Xin Zhou, Meng Yang, Jing Jin, Jie Chen.
